# Risk factors of contrast-associated acute kidneyinjury in spanish intensive care patients: preliminary results of the nefrocon study

**DOI:** 10.1186/2197-425X-3-S1-A53

**Published:** 2015-10-01

**Authors:** S Mas-Font, C Gómez-Gonzalez, D Herrera-Rojas, M Herrera-Gutierrez, L Del Baño-Aledo, MJ Broch-Porcar, E Moreno-Clarí

**Affiliations:** Hospital General Universitario de Castellón, Intensive Care Unit, Castellón, Spain; Hospital Infanta Luisa, Intensive Care Unit, Sevilla, Spain; Hospital Universitario de Valme, Intensive Care Unit, Sevilla, Spain; Complejo Universitario Carlos Haya, Intensive Care Unit, Málaga, Spain; Hospital Morales Meseguer, Intensive Care Unit, Murcia, Spain; Hospital Universitari i Politècnic La Fe, Intensive Care Unit, Valencia, Spain; Hospital Universitario General de Castellón, Intensive Care Unit, Castellón, Spain

## Objectives

It is essential to Know the profile of the patients at risk of developing contrast-associated acute kidney injury (CA-AKI) in order to define strategies for prevention. The aim of this study is to determine risk factors related to contrast-associated acute kidney injury in critical patients.

## Methods

We performed a prospective multi-center study, in 34 Spanish ICU, covering the period from 15 December 2012 to 15 March 2013. During this study period, we included 1035 patients, all of them undergoing a radiographic examination or a coronary angiography with administration of parenteral iodinated contrast media. We excluded patients with incomplete data or renal replacement at the time of the study, being finally 1012 patientes. We defined CA-AKI as an increase of serum creatinine ≥, 0,5 mg/dl, or ≥ 50 % from baseline, assessed 48-72 hours after the procedure. We performed the analysis using multiple logistic regression with CA-AKI as dependent variable using the R 3.1.2 software for OsX. We included in the analysis the variables with p < 0,1 in the univariate tests and those collected in the literature as related to the CA-AKI.

## Results

We find that the type of patient (p 0,001), age (p < 0,005), presence of risk background (p < 0,05) [ cirrhosis (p 0,003), heart failure (p < 0,005), previous transplant (p < 0,05), chronic kidney disease stage II-V (p < 0,001)], baseline serum creatinine (p < 0,001), APACHE II (p < 0,001), diuretics (p < 0,001), vasoactive therapy (p < 0,001) and shock (p < 0,001), were related to CA-AKI. CA-AKI was less frequent in coronary angiography (7,9 %) than in contrast enhanced TC (16,1 %) or other radiographic examinations (15,9 %, p < 0,001). Higher hemoglobin concentration (p < 0,001) at time of contrast administration was a protective factor. Diabetes, hypertension, use of metformin, and the type and volume of contrast are not shown as risk factors. Neither the application of preventive measures.

## Conclusions

In our cohort, severity of illness, previous kidney function and use of diuretics were the most relevant factors in relation to the development of CA-AKI, however, high levels of haemoglobin were as protectors, presenting lower incidence of CA-AKI.

**Table 1:** Multivariate analysis (test Hosmer- Lemeshow p 0,7.

	Coefficient	p	OR	95% CI
Apache II	,029	,045	1,030	1,001-1,060
Baseline serum creatinine	,389	,067	1,476	,973-2,238
Diuretics	,670	,003	1,955	1,247-3,065
Shock	,969	,000	2,636	1,634-4,253
Hemoglobin	-,114	,011	,892	,817-,974

